# Prevalence and Mortality Outcomes of Melioidosis in Thalassemia: A Systematic Review and Meta-Analysis

**DOI:** 10.3390/medsci13040216

**Published:** 2025-10-02

**Authors:** Jongkonnee Thanasai, Kritsada Singha, Atthaphong Phongphithakchai, Moragot Chatatikun, Sa-ngob Laklaeng, Jitabanjong Tangpong, Pakpoom Wongyikul, Phichayut Phinyo, Supphachoke Khemla, Anchalee Chittamma, Wiyada Kwanhian Klangbud

**Affiliations:** 1 Faculty of Medicine, Mahasarakham University, Mahasarakham 44000, Thailand; jongkonnee@msu.ac.th (J.T.); kritsada.si@msu.ac.th (K.S.); 2 Nephrology Unit, Division of Internal Medicine, Faculty of Medicine, Prince of Songkla University, Songkhla 90110, Thailand; atthaphong.p@psu.ac.th; 3 School of Allied Health Sciences, Walailak University, Nakhon Si Thammarat 80160, Thailand; moragot.ch@wu.ac.th (M.C.); sumoun2528@gmail.com (S.-n.L.); rjitbanj@wu.ac.th (J.T.); 4 Research Excellence Center for Innovation and Health Products (RECIHP), Walailak University, Nakhon Si Thammarat 80160, Thailand; 5 Center for Clinical Epidemiology and Clinical Statistics, Faculty of Medicine, Chiang Mai University, Chiang Mai 50200, Thailand; aumkidify@gmail.com (P.W.); phichayutphinyo@gmail.com (P.P.); 6 Department of Biomedical Informatics and Clinical Epidemiology (BioCE), Faculty of Medicine, Chiang Mai University, Chiang Mai 50200, Thailand; 7 Division of Infectious Diseases, Department of Internal Medicine, Nakhon Phanom Hospital, Nakhon Phanom 48000, Thailand; sup.mednkp@gmail.com; 8 Department of Pathology, Faculty of Medicine Ramathibodi Hospital, Mahidol University, Bangkok 10400, Thailand; anchalee.chi@mahidol.ac.th; 9 Medical Technology Program, Faculty of Science, Nakhon Phanom University, Nakhon Phanom 48000, Thailand

**Keywords:** melioidosis, *Burkholderia pseudomallei*, thalassemia, hemoglobinopathy, mortality, tropical infectious diseases, systematic review, meta-analysis

## Abstract

**Background.** Melioidosis is a severe infection caused by *Burkholderia pseudomallei* and is endemic in regions with a high prevalence of thalassemia. Patients with thalassemia are thought to be at increased risk due to iron overload, splenectomy, and immune dysfunction. However, the pooled prevalence and mortality outcomes of melioidosis in this population remain unclear. **Methods.** We conducted a systematic review and meta-analysis in accordance with PRISMA 2020 guidelines (PROSPERO: CRD420251108294). PubMed, Embase, and Scopus were searched from inception to July 2025. Observational studies reporting prevalence or mortality of melioidosis in patients with thalassemia were eligible. Pooled odds ratios (ORs) for mortality were calculated using random-effects models, with subgroup and sensitivity analyses based on age, thalassemia subtype, and study quality. **Results.** Six retrospective studies including 7529 melioidosis patients, of whom 173 had thalassemia, were analyzed. The prevalence of thalassemia among melioidosis cases ranged from 0.5% to 40.7%. Mortality among thalassemia patients varied from 0% to 100%. Pooled analysis demonstrated no significant excess mortality compared with non-thalassemia controls (OR 0.55, 95% CI 0.16–1.89; *I*^2^ = 44.9%). Sensitivity analysis restricted to moderate- and high-quality studies showed a significantly lower risk of death (OR 0.23, 95% CI 0.15–0.36; *I*^2^ = 0%). Subgroup analyses by thalassemia subtype and age revealed no clear effect modification, although power was limited. **Conclusions.** Despite biological plausibility, thalassemia was not associated with increased melioidosis mortality. These findings suggest that closer clinical monitoring, iron chelation, and comorbidity profiles may influence outcomes. Prospective, well-characterized cohort studies are needed to refine risk stratification and guide management in endemic regions.

## 1. Introduction

Melioidosis is a serious infectious disease caused by the environmental Gram-negative bacterium *Burkholderia pseudomallei*. The pathogen is endemic to tropical regions, particularly Southeast Asia and northern Australia, and is increasingly reported in South Asia, Africa, and South America [1,2]. Human infection occurs through percutaneous inoculation, inhalation, or ingestion of contaminated soil and water. The disease is characterized by a wide spectrum of clinical presentations, ranging from localized abscesses to pneumonia, septicemia, and multi-organ failure [3]. Despite improved awareness and the availability of antimicrobial therapy, mortality remains high, with case fatality rates often exceeding 40% in patients presenting with septic shock [3].

The global distribution of melioidosis overlaps substantially with regions of high thalassemia prevalence. Thalassemia comprises a group of inherited hemoglobinopathies characterized by defective globin chain synthesis, leading to chronic hemolytic anemia, ineffective erythropoiesis, and secondary complications such as iron overload [4,5]. The burden is particularly high in Southeast Asia, where carrier rates may reach 30–40% in some populations [4]. Patients with thalassemia are known to be predisposed to infections due to multiple mechanisms: chronic iron overload that enhances bacterial growth, splenectomy or splenic dysfunction that impairs clearance of pathogens, and neutrophil dysfunction leading to reduced host defense [6,7]. A range of bacterial pathogens, including *Salmonella*, *Yersinia*, and *Klebsiella*, are well-documented causes of severe infections in thalassemia [8]. However, the role of *B. pseudomallei* in this population has not been systematically evaluated.

Biological plausibility supports the hypothesis that thalassemia could increase susceptibility to severe melioidosis. Iron overload creates a favorable environment for *B. pseudomallei* growth [9,10], and impaired neutrophil function in thalassemia has been linked to increased vulnerability to bacterial infections [11]. Splenectomy, a common intervention in transfusion-dependent thalassemia, further increases infection risk [6]. However, other host and healthcare factors may counterbalance this risk. Regular follow-up, iron chelation therapy, and increased clinician awareness may contribute to earlier diagnosis and treatment, potentially reducing mortality. Moreover, comorbidities such as diabetes mellitus, a major risk factor for melioidosis, are less common in pediatric thalassemia populations compared to adults without thalassemia [12]. These factors may help explain the unexpected observation of lower mortality in some studies.

To date, no systematic synthesis of the available evidence has been conducted to clarify the relationship between thalassemia and melioidosis outcomes. Such evidence is essential for guiding clinical decision-making and public health planning. If thalassemia were confirmed to increase melioidosis risk or worsen outcomes, targeted preventive strategies, empirical antimicrobial choices, and closer clinical monitoring would be warranted. Conversely, if outcomes are not worse, assumptions of heightened risk in this group may need to be reconsidered.

Therefore, the objective of this systematic review and meta-analysis was to estimate the prevalence of thalassemia among melioidosis patients, to compare mortality outcomes between patients with and without thalassemia, and to explore potential effect modification by thalassemia subtype and age. By synthesizing data from multiple studies across endemic regions, we aimed to address a critical knowledge gap and provide evidence to inform risk stratification and clinical management strategies in areas where both diseases are highly prevalent.

## 2. Materials and Methods

### 2.1. Protocol and Registration

This systematic review and meta-analysis were conducted in accordance with the Preferred Reporting Items for Systematic Reviews and Meta-Analyses (PRISMA) 2020 statement [13]. The study protocol was prospectively registered in the International Prospective Register of Systematic Reviews (PROSPERO; registration number CRD420251108294).

### 2.2. Eligibility Criteria

We included observational studies (retrospective or prospective cohort studies, case–control studies, and case series with comparator groups) that reported clinical outcomes of melioidosis in patients with thalassemia. Eligible studies were required to provide data on prevalence, clinical characteristics, or mortality outcomes, with sufficient information to extract or calculate effect measures. We excluded review articles, conference abstracts, case reports without comparators, experimental or animal studies, and publications lacking outcome data relevant to thalassemia.

### 2.3. Information Sources and Search Strategy

A comprehensive literature search was conducted in PubMed, Embase, and Scopus from database inception to 1 July 2025. The search strategy combined controlled vocabulary and free-text terms related to thalassemia and melioidosis: (“thalassemia” OR “haemoglobinopathy” OR “hemoglobinopathy”) AND “melioidosis.” Detailed search strategies for each database are provided in Supplementary Table S1. Reference lists of eligible articles were screened manually to identify additional studies. Only studies published in English and involving human participants were considered.

### 2.4. Study Selection

Two reviewers (J.Thanasai and W.K.K.) independently screened titles and abstracts for relevance. Full texts of potentially eligible studies were then assessed against inclusion and exclusion criteria. Discrepancies were resolved by discussion or by consultation with a third reviewer. The study selection process is summarized in a PRISMA flow diagram.

### 2.5. Data Extraction

Two reviewers (J.Thanasai and W.K.K.) independently extracted data from each eligible study using a predesigned standardized template. The extracted information included details on study characteristics such as the first author, year of publication, country, and study design, as well as population characteristics including sample size, age distribution, and the presence of relevant comorbidities. For thalassemia-specific information, data were collected on thalassemia subtype, transfusion status, splenectomy history, and the use of iron chelation therapy when available. Outcomes of interest included the prevalence of thalassemia among melioidosis patients, clinical manifestations, and mortality rates in patients with thalassemia compared with non-thalassemia controls. Where provided, effect measures such as odds ratios (ORs) and risk ratios (RRs) were recorded directly; otherwise, raw data were extracted to allow calculation of effect estimates. Any disagreements between reviewers were resolved through discussion and consensus to ensure accuracy and completeness of the dataset.

### 2.6. Quality Assessment

The methodological quality of included studies was assessed using the Newcastle–Ottawa Scale (NOS), evaluating three domains: selection (maximum 4 points), comparability (maximum 2 points), and outcome (maximum 3 points). Two reviewers independently scored each study, with discrepancies resolved by discussion. Based on total scores, studies were categorized as low (0–3), moderate (4–6), or high quality (7–9).

### 2.7. Statistical Analysis

Pooled odds ratios (ORs) with 95% confidence intervals (CIs) were calculated to compare mortality outcomes between thalassemia and non-thalassemia patients with melioidosis. A random-effects model was applied to account for expected heterogeneity across studies. Statistical heterogeneity was quantified using the *I*^2^ statistic and τ^2^, with 95% CIs for heterogeneity obtained via the Q-profile method.

Subgroup analyses were conducted based on thalassemia subtype (β-thalassemia vs. unspecified) and age group (pediatric vs. adult populations). Sensitivity analyses were performed by excluding studies rated as low quality. Publication bias was not formally assessed because the small number of included studies (<10) and low event rates make funnel plot analysis and related tests unreliable [14].

All statistical analyses were performed using R software (R Foundation for Statistical Computing, Vienna, Austria), employing the meta package.

## 3. Results

### 3.1. Included Studies

**Figure 1:**
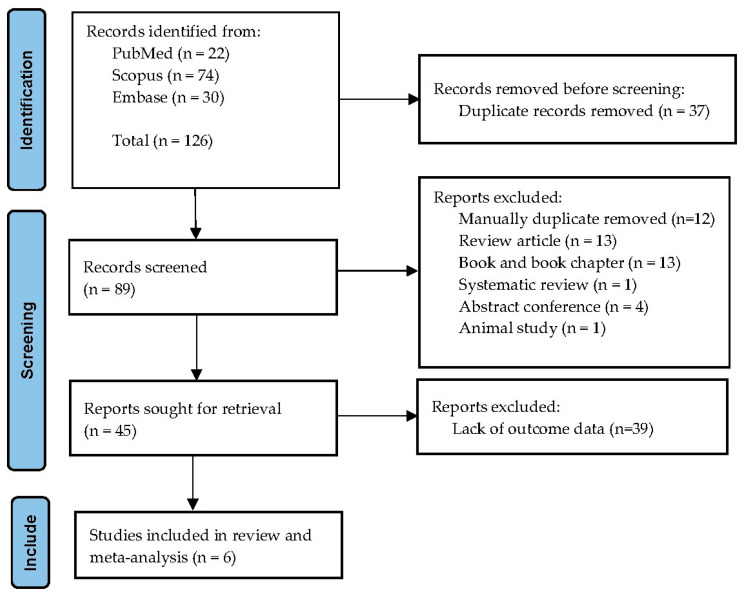
PRISMA flow diagram of study selection. The figure illustrates the number of records identified, screened, assessed for eligibility, and included in the final review and meta-analysis.

The database search retrieved 126 records: 22 from PubMed, 74 from Scopus, and 30 from Embase. After removal of 37 duplicates, 89 titles and abstracts were screened. Forty-four studies were excluded at this stage, mostly because they did not include thalassemia cases or lacked relevant outcome data. A total of 45 full-text articles were assessed, of which six fulfilled all inclusion criteria and were incorporated into the systematic review and meta-analysis. Reasons for exclusion of full texts included absence of comparator groups, case reports, or insufficient data for effect estimation. The overall selection process is illustrated in the PRISMA flow diagram (Figure 1).

### 3.2. Study Characteristics

Six retrospective studies including 7529 melioidosis patients, of whom 173 had thalassemia, were analyzed. They were conducted in five countries: Thailand, Malaysia, Cambodia, India, and Brunei. Sample sizes varied substantially. The largest was a nationwide retrospective Thai cohort comprising 7126 cases, while the smallest was a Malaysian pediatric series including only 27 patients. Other studies represented hospital-based cohorts or case series with intermediate sample sizes.

Thalassemia subtypes were inconsistently reported. Only two studies provided specific information: one confirmed β-thalassemia major in all pediatric patients, and another described post-splenectomy thalassemia cases, presumed to be transfusion-dependent. In most studies, thalassemia was recorded by diagnostic coding or medical records without genetic confirmation. The proportion of thalassemia among melioidosis cases varied markedly by setting. In India, only one case of thalassemia was reported among 199 patients (0.5%), while in a Malaysian pediatric series, nearly half of all cases (40.7%) occurred in children with thalassemia. Other reports ranged between these extremes, such as 2.1% in a nationwide Thai cohort and 5.2% in a southern Thai hospital-based cohort (Table 1). These variations reflect regional differences in both thalassemia prevalence and study design.

### 3.3. Study Quality

Quality assessment using the Newcastle–Ottawa Scale (NOS) revealed substantial heterogeneity. One large nationwide Thai study achieved a high score of 9/9, reflecting robust methodology and comprehensive data capture. Two studies were rated as moderate quality, typically due to limited detail on confounder adjustment or incomplete reporting of outcomes. Three smaller studies were rated as low quality, largely because of small sample sizes, retrospective design, and lack of clarity regarding outcome definitions. This variability in quality was taken into account during sensitivity analyses (Table 2).

### 3.4. Mortality Outcomes

Mortality among thalassemia patients with melioidosis varied widely between studies. In Malaysia, 36.4% of pediatric thalassemia patients with melioidosis died, compared with 75% of children without thalassemia, suggesting a relatively lower risk. In contrast, a Cambodian case series of 39 pediatric patients reported one fatal case, which occurred in the only child with thalassemia, resulting in a 100% case fatality within this subgroup. A southern Thai cohort of 134 patients included seven with thalassemia, none of whom died, compared with 9.5% mortality among non-thalassemia patients. The large Thai nationwide database showed lower in-hospital mortality in thalassemia compared with non-thalassemia patients (13.2% vs. 39.4%). Collectively, these findings demonstrate marked heterogeneity across settings and populations.

**Figure 2:**
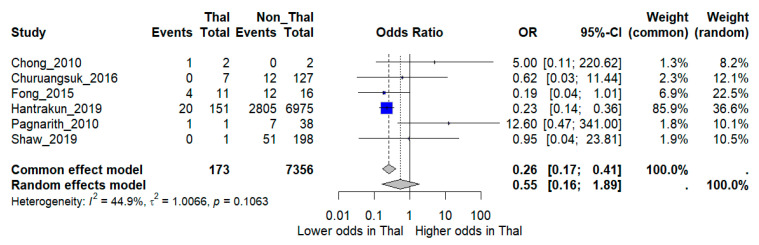
Forest plot of pooled odds ratios (OR) comparing mortality between melioidosis patients with thalassemia and those without thalassemia. Individual study estimates and the overall random-effects model are shown with 95% confidence intervals (CI). *I***^2^** indicates the percentage of total variation across studies due to heterogeneity. ***τ*^2^** represents the between-study variance. Statistical significance was defined as *p* < 0.05 [12,15,16,17,18,19].

When pooled in a random-effects meta-analysis, thalassemia was not associated with a statistically significant increase in mortality compared with non-thalassemia patients (OR 0.55, 95% CI 0.16–1.89). Between-study heterogeneity was moderate (*I*^2^ = 44.9%) (Figure 2).

### 3.5. Subgroup Analyses

**Figure 3:**
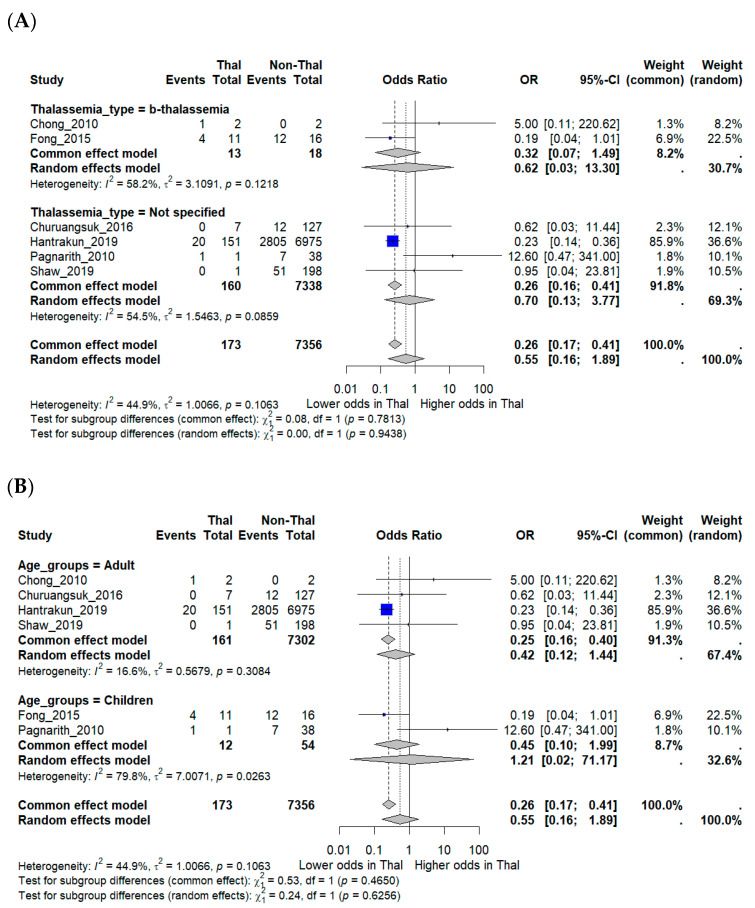
Subgroup analyses of mortality outcomes in melioidosis patients with thalassemia. (**A**) Subgroup analysis by thalassemia subtype (β-thalassemia vs. unspecified). (**B**) Subgroup analysis by patient age group (adults vs. children). Odds ratios (OR) with 95% confidence intervals (CI) are displayed. *I***^2^** indicates the percentage of total variation across studies due to heterogeneity. ***τ*^2^** represents the between-study variance. χ^2^ (Chi-square test) assesses whether differences across studies are due to chance. Statistical significance was defined as *p* < 0.05 [12,15,16,17,18,19].

Subgroup analyses were performed to explore potential effect modification. Studies that explicitly included β-thalassemia major did not show significantly different mortality compared with those that reported unspecified thalassemia. Age-stratified analyses suggested a trend toward lower mortality in adults (OR 0.42, 95% CI 0.12–1.44), while pediatric studies produced inconsistent findings, with one suggesting higher mortality but others showing little difference. Confidence intervals for pediatric estimates were wide (OR 1.21, 95% CI 0.02–71.17), reflecting the very small number of included cases and deaths. These limitations underscore the restricted statistical power of subgroup analyses (Figure 3A,B).

### 3.6. Sensitivity Analyses

**Figure 4:**
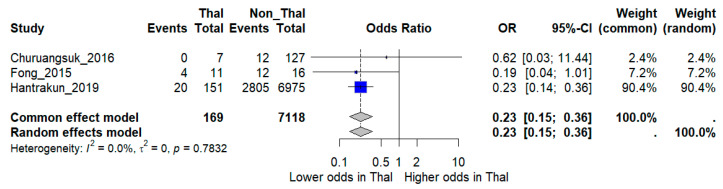
Sensitivity analysis excluding low-quality studies. Forest plot showing pooled odds ratios (OR) for mortality comparing thalassemia versus non-thalassemia patients, restricted to moderate- and high-quality studies. *I***^2^** indicates the percentage of total variation across studies due to heterogeneity. ***τ*^2^** represents the between-study variance. Statistical significance was defined as *p* < 0.05 [12,16,17].

Sensitivity analysis restricted to higher-quality studies provided more consistent results. When the three low-quality studies were excluded, the pooled analysis of three moderate- and high-quality studies comprising 7287 patients (~3000 deaths) demonstrated a significantly lower risk of death among thalassemia patients compared with non-thalassemia controls (OR 0.23, 95% CI 0.15–0.36). Notably, heterogeneity was eliminated in this restricted model (*I*^2^ = 0%), suggesting that methodological quality was a major source of inconsistency in the overall analysis (Figure 4).

## 4. Discussion

This systematic review and meta-analysis synthesized data from six observational studies including 7529 patients with culture-confirmed melioidosis, of whom 173 had thalassemia. Across studies, the prevalence of thalassemia among melioidosis cases varied widely, reflecting differences in regional thalassemia burden and study design. Mortality rates were equally heterogeneous, ranging from no deaths in some series to 100% mortality in small pediatric cohorts. When pooled, we found no evidence that thalassemia was associated with excess mortality. On the contrary, sensitivity analyses restricted to moderate- and high-quality studies suggested that thalassemia patients may have a lower risk of death. Although the pooled analysis suggested no excess mortality, this conclusion must be interpreted with caution due to extreme heterogeneity in prevalence and outcomes across studies. Furthermore, the comparator group consisted of all non-thalassemia melioidosis patients, who may themselves have had major risk factors such as uncontrolled diabetes or corticosteroid use. None of the included studies stratified outcomes by patients without identifiable comorbidities, limiting our ability to compare thalassemia cases with truly low-risk groups.

These findings diverge from the long-standing hypothesis that thalassemia predisposes to more severe melioidosis through iron overload, immune dysfunction, and splenectomy. Case reports and small pediatric series have described fulminant melioidosis in children with β-thalassemia major [9,17,18], supporting the notion of increased vulnerability. Earlier epidemiological studies also suggested thalassemia as a risk factor for melioidosis and bacteremic disease [9]. From a biological perspective, such associations are plausible since *B. pseudomallei* requires iron for growth [19,20], and iron overload is a hallmark of transfusion-dependent thalassemia. Impaired neutrophil function in thalassemia may further increase susceptibility [11].

However, our pooled estimates align more closely with recent large-scale data, particularly the nationwide Thai cohort, which demonstrated lower in-hospital mortality among melioidosis patients with thalassemia compared with those without [12]. Several explanations may account for this apparent paradox. Thalassemia patients often receive regular clinical monitoring in tertiary care centers, enabling earlier recognition and treatment of infections. The use of iron chelation therapy may reduce the adverse impact of iron overload on bacterial proliferation [19]. Furthermore, comorbidities such as diabetes mellitus—an established risk factor for poor melioidosis outcomes—are less common in pediatric thalassemia populations compared with adults without thalassemia [12]. These healthcare- and host-related factors may offset the biological disadvantages conferred by thalassemia.

These hypotheses remain speculative, however, as the included studies lacked detailed stratification by thalassemia subtype, transfusion status, or iron chelation therapy. Importantly, grouping β-thalassemia major together with minor or unspecified forms may have diluted a potential effect, since clinical management and prognosis differ substantially between these subtypes.

Nevertheless, several limitations must be acknowledged when interpreting these findings. The number of eligible studies was small, and most were retrospective with limited adjustment for confounders, reducing the certainty of pooled estimates. Thalassemia subtypes and disease characteristics were inconsistently reported, and information on transfusion status, iron chelation therapy, or splenectomy history was frequently absent, which precluded detailed subgroup analyses. Considerable heterogeneity in study design, sample size, and patient demographics (pediatric vs. adult, hospital-based vs. national cohorts) may also have contributed to variability in results. Mortality definitions were not standardized across studies, ranging from in-hospital to 30-day or crude fatality, which may have introduced outcome measurement bias. Selection bias is another concern, as many reports were from tertiary centers where thalassemia patients may benefit from earlier diagnosis and specialized care. Another key limitation is that >94% of patients originated from a single nationwide Thai cohort. As a result, the pooled findings may primarily reflect the Thai healthcare context and may not generalize to other endemic regions such as South Asia, Australia, or Africa, where clinical practices and pathogen epidemiology may differ. This overrepresentation raises the potential for selection bias. Finally, formal assessment of publication bias was not feasible due to the small number of studies, and standard methods such as funnel plots are considered unreliable in this context [14].

Despite these limitations, this review provides the first pooled evidence on melioidosis in thalassemia and challenges the assumption that this condition universally predisposes to worse outcomes. Future prospective, multicenter studies with standardized definitions, detailed clinical phenotyping, and inclusion of diverse endemic regions are essential to validate these findings, clarify underlying mechanisms, and refine risk stratification strategies.

## 5. Conclusions

This systematic review and meta-analysis found no evidence that thalassemia confers increased mortality in melioidosis. In contrast, sensitivity analyses restricted to higher-quality studies suggested a lower risk of death among thalassemia patients compared with non-thalassemia controls. These findings challenge the conventional assumption that thalassemia uniformly predisposes to worse infectious outcomes and underscore the importance of considering comorbidities, healthcare access, and iron management in determining prognosis. Future multicenter prospective studies with detailed clinical phenotyping are required to validate these results, clarify underlying mechanisms, and refine risk stratification strategies in endemic regions.

## Figures and Tables

**Table 1:** Characteristics of the six included studies on melioidosis in patients with thalassemia. Data include country, study design, population, thalassemia type, outcome measures, and mortality rates in cases and controls.

Author (Year) [Ref.]	Country	Design	Population	Thalassemia Case	Thalassemia Type	Outcome Measure	Mortality of the Thalassemia Case	Mortality of the Control Case	Note
Chong (2010) [15]	Brunei Darussalam	Retrospective case series	4 pancreatic melioidosis cases with CT scan	2 of 4	Post-splenectomy thalassemia	Pancreatic involvement, abscess resolution, mortality	1/2 (50%) with pancreatic melioidosis	0/2 (0%)	First report of pancreatic melioidosis; all abscesses resolved without drainage
Churuangsuk (2016) [16]	Thailand (South)	Retrospective 10-year hospital-based study	134 melioidosis patients	7 of 134	Not specified	Mortality, organ involvement	0/7 (0%)	12/127 (9.5%)	Thalassemia not associated with increased mortality
Fong (2015) [17]	Malaysia (Sabah)	Retrospective observational study	27 (Children < 15 years with culture-confirmed melioidosis)	11 of 27	β-thalassemia major	Incidence, bacteremia, organ dysfunction, mortality	4/11 (36.4%)	12/16 (75%)	Iron chelation reduced incidence; 59% overall mortality; high CNS involvement
Hantrakun (2019) [12]	Thailand (Nationwide)	Retrospective database study	7126 culture-confirmed melioidosis cases	151 of 7126	Not specified (ICD-10 code D56.0–D56.4, D56.8–D56.9)	30-day mortality	20/151 (13.2%)	2805/7126 (39.4%)	Thalassemia associated with significantly lower in-hospital mortality (aOR 0.53, 95% CI: 0.32–0.90, *p* = 0.02)
Pagnarith (2010) [18]	Cambodia	Retrospective case series	39 children with melioidosis	1 (with renal disease) of 39	Not specified	Mortality, clinical features	1/1 (100%)	7/38 (18.4%)	First pediatric report; 21% overall mortality
Shaw (2019) [19]	India (Southwest)	Retrospective molecular-epidemiology study	199 culture-confirmed melioidosis cases (Age 47.7 ± 15.4 years, range 7–86 years)	1 of 199	Not specified	Virulence genes, mortality	0/1 (0%)	51/198 (25.8%)	Thalassemia not associated with mortality

ICD-10: International Statistical Classification of Diseases and Related Health Problems, Tenth Revision. D56.0: α-thalassemia. D56.1: β-thalassemia. D56.2: thalassemia, unspecified. D56.3: iron deficiency in thalassemia. D56.4: hereditary persistence of fetal hemoglobin [HPFH]. D56.8: other thalassemia. D56.9: thalassemia, unspecified. aOR: adjusted odd ratio. CI: confidence interval. CT scan: computed Tomography scan.

**Table 2:** Quality assessment of included studies using the Newcastle–Ottawa Scale (NOS). Scores are presented for selection, comparability, and outcome domains, with total scores and quality ratings (low, moderate, high).

Study (Year)	[Ref.]	Selection (Max 4)	Comparability (Max 2)	Outcome (Max 3)	Total Score	Quality Level
Chong (2010)	[15]	2	0	2	4	Low
Churuangsuk (2016)	[16]	3	1	2	6	Moderate
Fong (2015)	[17]	3	1	2	6	Moderate
Hantrakun (2019)	[12]	4	2	3	9	High
Pagnarith (2010)	[18]	2	0	1	3	Low
Shaw (2019)	[19]	2	0	1	3	Low

## Data Availability

No new data were created.

## Supplementary Materials

The following supporting information can be downloaded at: https://www.mdpi.com/article/10.3390/medsci13040216/s1, Supplementary Table S1. Search strategies for PubMed, Embase, and Scopus from database inception to 1 July 2025.

## Abbreviations

The following abbreviations are used in this manuscript:

aOR	Adjusted odd ratio
CI	Confidence interval
CT	Computed Tomography
