# Supplementary material for: Prevalence and Mortality Outcomes of Melioidosis in Thalassemia: A Systematic Review and Meta-Analysis
Source: Med Sci (Basel). 2025 Oct 2;13(4):216. doi: 10.3390/medsci13040216 (PMC12551098; doi:10.3390/medsci13040216)
Supplement: Supplementary file 1 [file medsci-13-00216-s001.zip › medsci-3880700-supplementary.pdf]

medical sciences

MDPI

Prevalence and Mortality Outcomes of Melioidosis in Thalassemia: A Systematic Review and Meta-analysis

Jongkonnee Thanasai [1], Kritsada Singha [1], Atthaphong Phongphithakchai [2], Moragot Chatatikun [3,4], Sa-ngob Laklaeng [3], Jitabanjong Tangpong [3,4], Pakpoom Wongyikul [5,6], Phichayut Phinyo [5,6], Supphachoke Khemla [7], Anchalee Chittamma [8] and Wiyada Kwanhian Klangbud [9,*]

[1] Faculty of Medicine, Mahasarakham University, Mahasarakham 44000, Thailand
[2] Nephrology Unit, Division of Internal Medicine, Faculty of Medicine, Prince of Songkla University, Songkhla 90110, Thailand
[3] School of Allied Health Sciences, Walailak University, Nakhon Si Thammarat 80160, Thailand
[4] Research Excellence Center for Innovation and Health Products (RECIHP), Walailak University, Nakhon Si Thammarat 80160, Thailand
[5] Center for Clinical Epidemiology and Clinical Statistics, Faculty of Medicine, Chiang Mai University, Chiang Mai 50200, Thailand
[6] Department of Biomedical Informatics and Clinical Epidemiology (BioCE), Faculty of Medicine, Chiang Mai University, Chiang Mai 50200, Thailand
[7] Division of Infectious Diseases, Department of Internal Medicine, Nakhon Phanom Hospital, Nakhon Phanom 48000, Thailand
[8] Department of Pathology, Faculty of Medicine Ramathibodi Hospital, Mahidol University, Bangkok 10400, Thailand
[9] Medical Technology Program, Faculty of Science, Nakhon Phanom University, Nakhon Phanom 48000, Thailand
* Corresponding author E-mail: wiyadakwanhian@gmail.com

Supplementary Table S1:

Search strategies for PubMed, Embase, and Scopus from database inception to July 1, 2025.

Search no.	Query	Search Details	Results
**PubMed**			
#1	melioidosis [Title/Abstract] AND (thalassemia[Title/Abstract] OR hemoglobin[Title/Abstract] OR haemoglobin [Title/Abstract])	"melioidosis"[Title/Abstract] AND ("thalassemia"[Title/Abstract] OR "hemoglobin"[Title/Abstract] OR "haemoglobin"[Title/Abstract])	22
**EMBASE**			

 https://doi.org/10.3390/medsci13040216

#1	(meliodosis:ab OR 'melioidosis':ti) AND (thalassemia:ab OR thalassemia:ti OR hemoglobin:ab OR hemoglobin:ti OR haemoglobin:ab OR haemoglobin:ti)		32
**SCOPUS**			
#1	melioidosis AND thalassemia AND (LIMIT-TO (LANGUAGE , "English")) AND (LIMIT-TO (DOCTYPE , "ar"))		74
